# Efficacy and Safety of Sitagliptin in Japanese Patients With Type 2 Diabetes

**DOI:** 10.14740/jocmr1983w

**Published:** 2015-02-09

**Authors:** Hirotoshi Ohmura, Tomoya Mita, Yoshinobu Taneda, Masahiro Sugawara, Hideaki Funayama, Joe Matsuoka, Hirotaka Watada, Hiroyuki Daida, SPIRITS-J Study Investigators

**Affiliations:** a Department of Cardiovascular Medicine, Juntendo University Graduate School of Medicine, Tokyo 113-8421, Japan; b Department of Metabolism & Endocrinology, Juntendo University Graduate School of Medicine, Tokyo 113-8421, Japan; c Taneda Clinic, Iwaki, Fukushima 973-8402, Japan; d Sugawara Medical Clinic, Tokyo 177-0041, Japan; e Funayama Clinic, Tokyo 135-0047, Japan; f Department Center for Lifetime Cancer Education, Juntendo University Graduate School of Medicine, Tokyo 113-0033, Japan; g Sitagliptin Registration Type2 Diabetes-Juntendo Collaborating Project

**Keywords:** DPP-4 inhibitor, Treatment efficacy, Treatment safety

## Abstract

**Background:**

The aim of this study was to investigate the clinical efficacy and safety of sitagliptin in Japanese patients with type 2 diabetes.

**Methods:**

A total of 3,247 subjects treated with sitagliptin were retrospectively recruited. Glucose parameters were collected at baseline, and 1, 3 and 6 months after initiation of sitagliptin. In addition, we explored factors that can be used to predict sitagliptin-induced reduction in HbA1c using linear mixed effect model. Factors associated with hypoglycemic events were examined by logistic analyses.

**Results:**

We analyzed the available data of 3,201 subjects (1,287 females). Treatment of sitagliptin significantly reduced HbA1c level from 7.44±1.20% at baseline to 6.73±0.99% at 6 months (P < 0.0001). Linear mixed effect model analyses demonstrated that reduction of HbA1c was associated with higher baseline HbA1c level, younger age, lower BMI and sitagliptin monotherapy. During this study, 82 cases of hypoglycemia were recorded. Logistic analyses indicated that hypoglycemic events were more frequent in female patients, and patients with low BMI, long history of type 2 diabetes, high HbA1c and on combination therapy experienced. Other adverse events were rare and mild.

**Conclusions:**

Sitagliptin is effective for diabetic management and generally well tolerated in Japanese patients with type 2 diabetes. This trial was registered with UMIN (no. 000004121).

## Introduction

The prevalence of type 2 diabetes is increasing worldwide and 8.9 million Japanese individuals are estimated to suffer from type 2 diabetes [1]. Optimal management of type 2 diabetes by pharmacological interventions in addition to life style modification is necessary in order to achieve and maintain individualized glycemic goals that eventually reduce complications, morbidity and mortality [2]. Type 2 diabetes is a metabolic disorder characterized by a decline in insulin secretion and insulin resistance. Oral hypoglycemic agents (OHAs) such as sulphonylureas (SUs) and glinides promote insulin secretion and insulin therapy complement insulin secretion, while biguanide and thiazolidine improve insulin resistance. Although these drugs are widely used and effective in improving hyperglycemia, it is often difficult to maintain long-term glycemic control. This is partly due to the treatment-limiting side effects, such as hypoglycemia, weight gain, gastrointestinal symptoms and edema in addition to the natural progression of disease [3].

Sitagliptin, the first of a new class of OHA, dipeptidyl peptidase-4 (DPP-4) inhibitor, was first released in Japan in December 2009. Sitagliptin is a highly selective and reversible inhibitor of DPP-4[4]. It produces approximately two- to three-fold increases in active glucagon-like peptide-1 (GLP-1) and glucose-dependent insulinotropic polypeptide (GIP) levels [4], which stimulate glucose-dependent insulin response [5-7]. Previous studies suggested that monotherapy of once-daily 100 mg sitagliptin for 12 weeks significantly reduced HbA1c by 0.65% in Japanese patients with type 2 diabetes [8], consistent with the findings reported in non-Japanese subjects [4]. Based on its mechanism of action, sitagliptin monotherapy is associated with only few episodes of hypoglycemia and does not produce significant body weight gain [8]. In this regard, while a limited prescribers’ group in Japan reported no episode of serious hypoglycemia during clinical trials of the combination of sitagliptin and SU [9], in daily clinical setting, serious and frequent hypoglycemic adverse events (AEs) were reported especially in elderly patients with renal dysfunction treated with DPP-4 inhibitors combined with high-dose SU [10]. The committee regarding to adequate use for incretin-based therapy advocates reduction of the dose of SU when used in combination with DPP-4 inhibitors [11]. Thus, it is important to investigate the efficacy and safety of DPP-4 inhibitors in patients with type 2 diabetes.

The present study was designed to evaluate the efficacy and safety of sitagliptin in a large number of Japanese patients with type 2 diabetes, and to identify those factors that affect glycemic control and hypoglycemic episodes.

## Methods and Design

### Subjects

This is a retrospective 6-month observational study of sitagliptin as add-on therapy to patients with type 2 diabetes treated by diet and exercise, OHA and/or insulin therapy. A total of 3,247 patients with type 2 diabetes were recruited in this cohort study at the Outpatient Clinic of Juntendo University Hospital, other satellite hospitals, and private clinics between January 2010 and March 2012. The follow-up period extended to October 2012. Diabetologists, cardiovascular specialists, hospital physicians and general practitioners participated in this study as investigators. We excluded patients with history of diabetic ketoacidosis or hyperosmolar non-ketonic coma within 6 months, infections, renal dysfunction (serum creatinine ≥ 13.26 μmol/dL), and pregnant or breast feeding females. Given the observational nature of the study, all procedures, including adjustment of sitagliptin dose during this study constituted the routine clinical care provided to the patients. The ethics committees of the participating hospitals approved the study protocol and informed consent was obtained from each subject. This study was registered with the clinical trial (UMIN 000004121).

### Data collection

Baseline demographic data were collected for all patients enrolled in this study. Data were collected around four time points: sitagliptin initiation, and 1, 3 and 6 months after sitagliptin initiation. The attending physicians also collected information from the patient’s recall and medical records.

### Study endpoints and assessments

The primary efficacy endpoint is the change in HbA1c from baseline to 6 months after initiation of sitagliptin. Secondary endpoints are changes in HbA1c from baseline to 1 and 3 months after initiation of sitagliptin, the proportion of patients achieving HbA1c (National Glycohemoglobin Standardization Program (NGSP)) < 7.0%, 6 months after initiation of sitagliptin, changes from baseline in lipid parameters to 1, 3 and 6 months after sitagliptin, and safety and tolerability during the study period. The latter were evaluated by reviewing investigator-reported AEs, physical examinations, vital signs, body weight and laboratory test results, including hematology, serum chemistry, and urinalysis. Severe hypoglycemia represented hypoglycemic episode that required the attention and assistance of another person. Another endpoint was to identify those factors that affect glycemic control.

### Biochemical tests

Blood test results were obtained from the medical records. Glucose and HbA1c were measured with standard techniques. The value of HbA1c (%) was estimated as the NGSP equivalent value (%) calculated by the formula HbA1c (%) = (HbA1c (Japan Diabetes Society) (%) + 0.4%) [12]. The estimated glomerular filtration rate (eGFR) was calculated by the following formula: eGFR (mL/min/1.73 m^2^) = 194 × Age^-0.287^ × Serum creatinine^-0.1094^ (× 0.739 for females) [13].

### Statistical analysis

Results are presented as mean ± SD or %. Repeated measurement analysis of variance (using mixed effect model) was used to evaluate the effect of 6-month sitagliptin treatment. P-value was calculated by type III tests of fixed effects. Differences between baseline data and any observation points were examined for statistical significance using the Student’s *t*-test. Categorical variables were compared using a Chi-square test, and presented as absolute frequencies with percentages.

The linear mixed effect model was used to determine the predictive value of certain baseline clinical parameters for sitagliptin-induced reduction of HbA1c. Age (< 65 vs. ≥ 65), gender (male vs. female), duration of type 2 diabetes (< 10 years vs. ≥ 10 years), BMI (< 25 vs. ≥ 25), HbA1c levels (6, vs. 6 - 6.9 vs. 7.0 - 7.9 vs. ≥ 8), eGFR (60, vs. ≥ 60) and type of anti-diabetes drugs (sitagliptin monotherapy vs. sitagliptin plus SU vs. sitagliptin plus OHAs except SU and/or insulin) were entered as covariates, which were normally distributed as identified by tests of normality.

Multiple logistic regression analysis was performed to identify factors that were independently correlated with hypoglycemic events; hypoglycemic events were set as the dependent variable while the independent variables included age (< 65 vs. ≥ 65), gender (male vs. female), duration of type 2 diabetes (< 10 years vs. ≥ 10 years), BMI (< 25 vs. ≥ 25), HbA1c levels (6, vs. 6 - 6.9 vs. 7.0 - 7.9 vs. ≥ 8), eGFR (60, vs. ≥ 60) and type of anti-diabetes drugs (sitagliptin monotherapy vs. sitagliptin plus SU vs. sitagliptin plus OHAs except SU and/or insulin). The models were fitted to the data using stepwise selection applied to an *a priori* model specification (set 0.3 and 0.35 as the significant level for entering and removing effects, respectively). Subsequently, P-values derived from maximum likelihood estimates, odds ratio (OR) estimates and Wald 95% confidence interval (CI) were calculated. Model results are presented as OR with 95% CIs. All analyses were performed using SAS ver.9.2 (SAS Institute Inc., Cary, NC, USA).

## Results

We enrolled 3,247 patients and collected 3,201 case report forms. Forty-six patients were excluded from analysis due to insufficient data which were defined that we could not collect any data at 1, 3 and 6 months except baseline. The clinical characteristics of the 3,201 study patients were as follows: the mean age was 65.0 ± 11.4 years, 53.2% were men, BMI was 25.1 ± 4.3 kg/m^2^, estimated duration of type 2 diabetes was 9.56 ± 7.58 years and HbA1c (NGSP) was 7.44±1.20% (Table 1). Twenty-four percent of patients received sitagliptin monotherapy and the rest of patients received combination therapy. The most common class of OHA concomitantly used was SUs (59.4%), followed by biguanide (45.5%), thiazolidine (23.4%), alpha-glucosidase inhibitors (13.8%), insulin (8.8%), and glinides (2.2%).

**Table 1:** Baseline Characteristics of Study Subjects (n = 3,201)

Baseline parameters	
Age (years) (n = 3,201)	65.0 ± 11.4
Gender (male/female) (n = 3,198)	1,911/1,287
Body mass index (kg/m^2^) (n = 2,845)	25.1 ± 4.3
Estimated duration of diabetes (years) (n = 2,914)	9.56 ± 7.58
HbA1c (NGSP) (%) (n = 3,132)	7.44 ± 1.20
Estimated glomerular filtration rate (mL/min/1.73 m^2^) (n = 2,702)	69.2 ± 47.6
Glucose lowering agents (%)	
Sulfonylurea	59.40%
Glinide	2.20%
α-glucosidase inhibitor	13.80%
Thiazolidinedione	23.40%
Metformin	45.50%
Insulin therapy	8.80%

Data are mean ± SD or number or % of subjects.

The mean dosages of sitagliptin significantly increased from 48.8 ± 7.5 mg/day at baseline to 52.5 ± 14.6 mg/day at 6 months (P < 0.05). Table 2 shows glycemic primary endpoint after the addition of sitagliptin. HbA1c decreased significantly from baseline to 1, 3 and 6 months after the initiation of sitagliptin. The majority of sitagliptin effect on HbA1c was achieved within 3 months after initiation of sitagliptin and was maintained for the next 3 months. Similarly, random blood glucose levels showed significant improvement from baseline to 1, 3 and 6 months after the initiation of sitagliptin. The proportion of patients who achieved HbA1c target of < 7.0% (NGSP) was 43.3% at 6 months after the initiation of sitagliptin, compared with 12.2 at baseline. On the other hand, no significant change in BMI was observed throughout the study.

**Table 2:** Changes in Clinical Parameters After Sitagliptin Treatment (n = 3,201)

	Baseline	At 1 month	At 3 months	At 6 months	P value*
Body mass index (kg/m^2^)	25.1 ± 4.3	25.2 ± 4.3	25.2 ± 4.3	25.6 ± 4.3	NS
n	2,845	1,937	2,026	1,512	
HbA1c (NGSP) (%)	7.44 ± 1.20	7.07 ± 1.03**	6.75 ± 0.92**	6.73 ± 0.99**	< 0.0001
n	3,132	2,848	2,959	2,946	
Random blood glucose level (mmo/L)	9.69 ± 3.56	8.42 ± 2.88**	8.32 ± 2.78**	8.47 ± 2.91**	< 0.0001
n	3,102	2,858	2,946	2,923	

Data are mean ± SD or number of subjects. *Changes in clinical parameters after sitagliptin treatment were evaluated by analysis of variance using mixed effects model. **Comparison between baseline value and each value at 1, 3 and 6 months, by Student’s *t*-test. P < 0.05. NGSP: National Glycohemoglobin Standardization Program.

To investigate whether reduction of HbA1c by sitagliptin could be predicted by baseline clinical parameters, we used the linear mixed effect model with candidate predictive factors such as age, gender, duration of type 2 diabetes, BMI, HbA1c level, eGFR and type of anti-diabetes drugs (sitagliptin monotherapy vs. sitagliptin plus SUs vs. sitagliptin plus OHAs except SUs and/or insulin) at baseline as independent variables. In these analyses, the most appropriate model was selected (Table 3). First, sitagliptin treatment significantly and progressively reduced HbA1c levels during the observation period. In addition, reduction of HbA1c correlated significantly with younger age, lower BMI, higher baseline HbA1c, and sitagliptin monotherapy, whereas there was no interaction between period and BMI (Table 3, and data not shown).

**Table 3:** Predictive Value of Baseline Clinical Parameters for HbA1c Reduction by Sitagliptin Using Linear Mixed Effect Model (Type III Tests of Fixed Effects) (n = 3,201)

Effect	Numerator of degree of freedom	Denominator of degree of freedom	F value	Pr > F
Treatment period	3	8,076	839.64	< 0.001
Treatment period × body mass index (kg/m^2^)	3	8,076	0.91	0.4330
Age (years)	1	3,071	9.29	0.0023
Gender (female/male)	1	3,071	0.07	0.7871
Body mass index (kg/m^2^)	1	3,071	4.44	0.0353
Estimated duration of diabetes (years)	1	3,071	2.42	0.1202
Baseline HbA1c levels (%) (NGSP)	3	3,071	1,512.15	< 0.001
eGFR (mL/min/1.73 m^2^)	1	3,071	1.94	0.1640
Type of diabetes drugs	2	3,071	7.90	0.0004

Age (< 65 vs. ≥ 65), gender (male vs. female), duration of type 2 diabetes (< 10 years vs. ≥ 10 years), BMI (< 25 vs. ≥ 25), HbA1c levels (6, vs. 6 - 6.9 vs. 7.0 - 7.9 vs. ≥ 8), eGFR (60, vs. ≥ 60) and type of anti-diabetes drugs (sitagliptin monotherapy vs. sitagliptin plus SU vs. sitagliptin plus OHA and/or insulin without SU) were entered as covariate. eGFR: estimated glomerular filtration rate; NGSP: National Glycohemoglobin Standardization Program.

AEs were observed in 323 patients. The most frequent AE was hypoglycemia, followed by constipation and dizziness (Table 4). Overall, AEs in 53 cases resulted in discontinuation of sitagliptin (1.7%). Hypoglycemia occurred in 82 patients. Among them, only two patients on SU or insulin therapy experienced severe hypoglycemia. Including these two events, only four patients among the 82 patients discontinued sitagliptin. The number of patients on monotherapy who experienced hypoglycemia was only two (2.4%), while a higher percentage of patients who received the combination of sitagliptin and SU or insulin tended to experience hypoglycemia (about 75% and 31%, respectively).

**Table 4:** Clinical and Laboratory Safety and Tolerability Results (n = 3,201)

	Number of patients with clinical adverse effects	Number of patients who discontinued due to adverse effects
Hypoglycemia	82 (2.5%)	4
Constipation	29 (0.90%)	7
Dizziness	25 (0.77%)	4
Eruption	24 (0.74%)	10
Edema	20 (0.62%)	1
Liver dysfunction	19 (0.59%)	5
Abdominal distension	18 (0.56%)	4
Diarrhea	10 (0.31%)	2
Abdominal pain	8 (0.25%)	1
Nausea and vomiting	7 (0.22%)	1
Appetite loss	3 (0.09%)	0
Others	78 (2.4%)	14
Total	323 (10.1%)	53

*Data are number of subjects (%).

Logistic regression analyses identified the type of anti-diabetes drugs, BMI, gender, duration of type 2 diabetes and HbA1c levels at baseline as significant predictors of frequent hypoglycemic events (Table 5). Hypoglycemic events were less frequent in patients on sitagliptin monotherapy compared to those on sitagliptin plus SUs or sitagliptin plus OHAs except SUs and/or insulin. Similarly, the frequency of hypoglycemic events was higher in female patients, and patients with lower BMI, longer duration of type 2 diabetes, and HbA1c more than 8.0% at baseline, compared with male patients, and patients with higher BMI, shorter duration of type 2 diabetes, and HbA1c less than 6.0%.

**Table 5:** Predictors of Hypoglycemia by Multiple Logistic Regression (n = 3,201)

Variables at baseline	Odds ratio (Wald 95% confidence interval)
Type of anti-diabetes drugs	
Sitagliptin plus SU vs. sitagliptin monotherapy	16.25 (7.633 - 34.61)
Sitagliptin plus OHA and/or insulin without SU vs. sitagliptin monotherapy	6.23 (2.75 - 14.36)
Female vs. male	1.47 (1.17 - 1.85)
Body mass index (< 25 vs. ≥ 25)	1.95 (1.52 - 2.50)
Estimated duration of diabetes (years) (≥ 10 vs. < 10)	1.28 (1.02 - 1.62)
Baseline HbA1c level (%) (NGSP)	
< 6 vs. ≥ 8	0.35 (0.13 - 0.96)
6.0 - 6.9 vs. ≥ 8	1.17 (0.87 - 1.58)
7.0 - 7.9 vs. ≥ 8	1.14 (0.85 - 1.52)

NGSP: National Glycohemoglobin Standardization Program.

The numbers of gastrointestinal symptoms including constipation, diarrhea, abdominal distension and abdominal pain were the second on the list of frequently observed AEs, although all the reported AEs were not severe. Skin eruption was reported in 24 patients and 10 cases were considered to be sitagliptin-related AEs because discontinuation of the drug improved symptoms. Increased alanine aminotransferase (ALT) or aspartate aminotransferase (AST) was found in 21 patients. In five of these cases, sitagliptin was discontinued. Especially, ALT or AST levels in two patients were more than three-fold higher than the upper limit of the normal range but returned to normal levels after discontinuation of sitagliptin. No other frequently observed or serious AEs were observed in this study.

## Discussion

The results of this retrospective study of 3,247 patients who received sitagliptin indicated that sitagliptin administration produced significant and clinically meaningful improvement of glycemic control and was generally well tolerated in Japanese patients with type 2 diabetes.

In the present study, sitagliptin significantly reduced HbA1c by about 0.7% for 6 months in Japanese patients with type 2 diabetes. Similar reductions in HbA1c were also reported in previous clinical studies [8, 9] and clinical observation studies conducted in Japan [14, 15]. It is of note that in the early stage of this study, α-glucosidase inhibitors and glinides were frequently switched to sitagliptin, because the health insurance system in Japan did not allow the combination of sitagliptin and α-glucosidase inhibitors or glinides at that time. Indeed, the proportions of patients who received the combination of α-glucosidase inhibitors or glinides were very low (13.8% and 2.2%, respectively, Table 1). Given that significant improvements in glycemic parameters were especially observed in Japanese patients with type 2 diabetes treated by sitagliptin combined with α-glucosidase inhibitors [16], switching these drugs to sitagliptin may underestimate potential glucose-lowering effect of sitagliptin. In addition, we could not collect detailed data about adjustment in other OHA in this study. Especially, investigators tended to reduce the SU dose based on safety alert by the committee regarding to adequate use for incretin-based therapy [11], which also underestimated the glucose-lowering effect of sitagliptin.

The present study found that HbA1c reduction was associated with baseline high HbA1c level, young age, low BMI and sitagliptin monotherapy using the linear mixed effect model. In general, patients with higher HbA1c levels were likely to achieve greater reductions in HbA1c levels. In fact, previous studies reported that baseline HbA1c was an important predictor of the efficacy of not only sitagliptin [14, 15] but also other OHAs [17].

In the natural history of type 2 diabetes, beta cell function is known to deteriorate gradually [18], thus, younger patients tend to have relatively preserved beta cell function. Thus, young age as a predictor of reduction of HbA1c level might be related to the preserved beta cell function. Indeed, it was shown that DPP-4 inhibitors were effective in Japanese type 2 diabetes patients with higher insulin secreting capacities [19]. Theoretically, patients with short history of type 2 diabetes also have preserved beta cell function. Previous reports demonstrated that short history of type 2 diabetes was associated with greater reductions of HbA1c by DPP-4 inhibitors [14, 15, 20]. In this study, HbA1c reduction did not correlate with the duration of type 2 diabetes by linear mixed effect model. In this regard, several studies did not show any relationships between glucose-lowering effect of sitagliptin and duration of type 2 diabetes [21, 22]. Increased GLP-1 by DPP-4 inhibitors lowers glucose not only by enhancing insulin secretion but also by suppressing glucagon secretion. These effects were likely to almost equally contribute to glucose lowering in patients with type 2 diabetes [23]. Accordingly, suppressive effect of glucagon by DPP-4 inhibitor may mainly contribute to glucose lowering in longer duration of type 2 diabetes patients with decreased insulin secretion capacities.

Lower BMI was another independent predictor of the efficacy of sitagliptin similar to previous studies in Japanese patients with type 2 diabetes [14, 15, 24]. Recently, a meta-analysis showed that BMI was a predictor of the glucose lowering effect of DPP-4 inhibitors even among distinct ethnic groups. Their efficacy was higher in Asians, because BMI of Asian type 2 diabetes is lower than other ethnic groups [25]. Why higher BMI is associated with a smaller sitagliptin-induced reduction in HbA1c remains largely unknown. However, one recent study demonstrated that serum soluble DPP-4 levels were significantly higher in obese subjects than lean subjects, probably by DPP-4 release from adipose tissue, depending on its volume [26]. Another study reported that high serum levels of soluble DPP-4 correlated with poor response to sitagliptin in Japanese patients with type 2 diabetes [27]. Taken together, serum soluble DPP-4 levels could be related to responsiveness of sitagliptin depending on BMI. Furthermore, given that GLP-1 levels in response to oral intake are reduced in obese patients [28, 29], the capacity of GLP-1 secretion might be also involved in the responsiveness of sitagliptin depending on BMI.

Sitagliptin has been demonstrated to improve glycemic control when added to SUs [9], metformin [30], pioglitazone [31], and insulin therapy [20, 32] in Japanese patients with type 2 diabetes. We also confirmed that the combination of sitagliptin and other OHA and/or insulin significantly improved glycemic control (data not shown). However, in this study, the reduction in HbA1c with sitagliptin monotherapy was greater than those in sitagliptin-SU group and sitagliptin-other OHA and/or insulin group. This may be due to the fact that patients with sitagliptin monotherapy represented early stage of type 2 diabetes that has preserved β cell function. In fact, patients on sitagliptin monotherapy had the shortest history of type 2 diabetes among groups (8.66 years for sitagliptin monotherapy, 11.8 years for sitagliptin-SU group and 11.2 years in sitagliptin-other OHA and/or insulin group).

Treatment-induced hypoglycemia is considered a major concern in the management of patients with type 2 diabetes. In this study, the incidence of hypoglycemia seemed to be low similar to previous clinical studies [8, 30, 31, 33-36]. Generally, it was reported that the use of glucose-lowering agents, old age, renal dysfunction, long duration of type 2 diabetes, aggressive therapy and obesity increased hypoglycemic events [37, 38]. Consistent with these findings, logistic analysis identified longer history of type 2 diabetes and combination therapy at baseline correlated significantly with hypoglycemic events in this study. With regard to glucose lowering agents, SU and insulin therapy are regarded as factors related to hypoglycemic events. Indeed, some clinical studies demonstrated that hypoglycemic episodes were especially frequent when DPP-4 inhibitors were used in combination with SU and insulin therapy. The reported incidence of hypoglycemia was 5.6-10% in sitagliptin-SU combination therapy [9, 39] and 16-20.2% [32, 40] in sitagliptin-insulin combination. In contrast, we found lower incidence of hypoglycemia even in patients treated with sitagliptin-SU and sitagliptin-insulin, although the incidence was higher in dual therapy than in sitagliptin monotherapy. The reason for the lower incidence was likely to be due to the allowance of adjustment of other OHA dose or insulin dose over the course of study.

Other factors that showed significant relationship with hypoglycemic events were low BMI and high HbA1c levels at baseline. Since we showed greater reductions in HbA1c in patients with low BMI and high HbA1c levels in this study, this result is reasonable. On the other hand, we also found that female gender correlated with hypoglycemic events. Although the exact reason for this finding is not clear, this finding is similar to that of a recent study which reported a similar association between female gender and higher risk of severe hypoglycemia [41].

All other clinical and laboratory AEs recorded in the present study were not serious and their incidence was low. Thus, sitagliptin should be well tolerated in clinical practice, similar to preclinical studies in Japan [8, 9, 32] and a previous pooled analysis of 19 double-blind clinical studies conducted by Merk & Co., Inc. [42]. The associations between OHA and cardiovascular disease and between OHA and malignancy have been recently the focus of increased attention. Especially, the US Food and Drug Administration (FDA) has issued guidance advising development programmers to indicate that all new OHAs do not have undesirable effect on cardiovascular events. Recent studies reported that the rates of cardiovascular diseases were not increased with DPP-4 inhibitors [43, 44]. However, long-term assessment of the efficacy and safety of the drug is essential for proper evaluation of the drug. In this regard, we are planning longer-term follow-up study that focuses on the efficacy and safety of sitagliptin treatment in the same cohort.

Our study has certain limitations. First, the study was a retrospective observation study without a control arm, which may cause a selection bias. Therefore, the results of this study must be interpreted with caution in comparison with other studies. Second, there was little information on life style, including diet and exercise therapy and drug compliance and confounding effects by other medications. Furthermore, lack of information on proportion and dosages of OHAs and insulin at 6 months were major limitations to interpret the results. Also, there were some missing values in several variables. Especially, change in BMI was important to access drug effects in management of diabetes. Third, the safety and tolerability were mainly evaluated by reviewing investigator-reported AEs, which may influence the number of AEs. Fourth, the measurement methods of laboratory data were not standardized across the study.

In conclusion, the results of the present study suggested that treatment with sitagliptin resulted in a significant and clinically meaningful improvement in glycemic control and was generally well tolerated in patients with type 2 diabetes in clinical practice.

## Abbreviations

AEs: adverse events
ALT: alanine aminotransferase
ANCOVA: analysis of covariance
AST: aspartate aminotransferase
BMI: body mass index
CI: confidence interval
DPP-4: dipeptidyl peptidase-4
eGFR: estimated glomerular filtration rate
FDA: US Food and Drug Administration
GIP: glucose-dependent insulinotropic polypeptide
GLP-1: glucagon-like peptide-1
NGSP: National Glycohemoglobin Standardization Program
OHA: oral hypoglycemic agent
OR: odds ratio
SU: sulphonylurea
